# Extracellular vesicles from triple-negative breast cancer cells promote proliferation and drug resistance in non-tumorigenic breast cells

**DOI:** 10.1007/s10549-018-4925-5

**Published:** 2018-09-01

**Authors:** Patricia Midori Murobushi Ozawa, Faris Alkhilaiwi, Iglenir João Cavalli, Danielle Malheiros, Enilze Maria de Souza Fonseca Ribeiro, Luciane Regina Cavalli

**Affiliations:** 1 Department of Oncology, Lombardi Comprehensive Cancer Center, Georgetown University Medical Center, Washington, DC USA; 2 Department of Genetics, Federal University of Paraná, Curitiba, PR Brazil; 3 Faculty of Pharmacy, King Abdulaziz University, Jeddah, Saudi Arabia

**Keywords:** TNBC, Exosomes, Proliferation, Drug resistance, Gene expression, miRNA expression

## Abstract

**Purpose:**

Triple-negative breast cancer (TNBC), an aggressive breast cancer subtype, is genetically heterogeneous which challenges the identification of clinically effective molecular makers. Extracellular vesicles (EVs) are key players in the intercellular signaling communication and have been shown to be involved in tumorigenesis. The main goal of this study was to evaluate the role and mechanisms of EVs derived from TNBC cells in modulating proliferation and cytotoxicity to chemotherapeutic agents in non-tumorigenic breast cells (MCF10A).

**Methods:**

EVs were isolated from TNBC cell lines and characterized by nanoparticle tracking analysis, Western blot, and transmission electron microscopy. MCF10A cells were treated with the isolated EVs and evaluated for cell proliferation and cytotoxicity to Docetaxel and Doxorubicin by the MTT and MTS assays, respectively. Gene and miRNA expression profiling was performed in the treated cells to determine expression changes that may be caused by EVs treatment.

**Results:**

MCF10A cells treated with HCC1806-EVs (MCF10A/HCC1806-EVs) showed a significant increase in cell proliferation and resistance to the therapeutic agents tested. No significant effects were observed in the MCF10A cells treated with EVs derived from MDA-MB-231 cells. Gene and miRNA expression profiling revealed 138 genes and 70 miRNAs significantly differentially expressed among the MCF10A/HCC1806-EVs and the untreated MCF10A cells, affecting mostly the PI3K/AKT, MAPK, and HIF1A pathways.

**Conclusion:**

EVs isolated from the HCC1806 TNBC cells are capable of inducing proliferation and drug resistance on the non-tumorigenic MCF10A breast cells, potentially mediated by changes in genes and miRNAs expression associated with cell proliferation, apoptosis, invasion, and migration.

**Electronic supplementary material:**

The online version of this article (10.1007/s10549-018-4925-5) contains supplementary material, which is available to authorized users.

## Introduction

Triple-negative breast cancer (TNBC) is an aggressive subtype of breast cancer, characterized by high proliferation rates and by conferring poor overall survival to the patients [22]. These tumors are molecularly heterogeneous [25, 32] which challenges the identification of effective prognostic molecular markers and target therapies.

Exosomes are extracellular vesicles (EVs) of endocytic origin that are present in body fluids and are known to play key roles in intercellular signaling communication [9, 26, 33]. A continuous dialog between tumor and stromal cells is essential to tumor development, and EVs have been described as tumor mediators responsible to modulate tumor-stromal cells signaling [4]. The EVs’ effects on tumorigenesis seem to occur in a cell of origin dependent manner, indicating that the treated cells acquire characteristics that resemble the EVs cell of origin [4]. Several studies have shown the involvement of EVs in modulating several cancer phenotypes [16], including immune suppression [5], angiogenesis [14, 34], cell migration [17], tumor invasion [12, 30], and drug resistance [3, 39], highlighting their relevance to tumorigenesis [11]. However, there are few reports that describe the role of TNBC-derived EVs in cancer progression [1, 13, 21, 23], and their actual mechanism and tumorigenic cellular effects remain unknown.

Therefore, the aim of this study was to evaluate the tumorigenic effects of EVs derived from TNBC cells, when co-cultivated with the non-tumorigenic breast cells MCF10A. The TNBC-EVs’ effects in these cells were measured by evaluating cell proliferation and cytotoxicity to chemotherapeutic agents and their corresponding alterations in gene and miRNA expression patterns.

## Materials and methods

### Cell culture

Two TNBC cell lines, HCC1806 and MDA-MB-231, were used to determine the effects of EVs in the MCF10A non-tumorigenic cells. The MCF-7 cell line (luminal A subtype) was used as a control for the TNBC specificity of the EVs effects. MCF10A, MCF-7, and MDA-MB-231 cell lines were obtained from the Tissue Culture Shared Resource (TCSR), Lombardi Comprehensive Cancer Center (LCCC), Georgetown University, USA. The HCC1806 was gently donated by Dr. Riggins from LCCC. MCF10A cells were cultivated in DMEM/F12 media (Gibco) with 2.5 mM l-glutamine, 20 ng/ml epidermal growth factor, 0.01 mg/ml insulin, 500 ng/ml hydrocortisone, and 5% horse serum. MCF-7, MDA-MB-231, and HCC1806 cell lines were cultivated in RPMI 1640 media (Gibco) with 10% fetal bovine serum (FBS) and 0.5% of penicillin–streptomycin. Cells were cultured with 5% CO_2_ at 37 °C. FBS exosome-depleted media (Gibco) were used (EV media) for EV isolation and functional assays.

### EVs isolation and characterization

For all the breast cell lines studied, the EV media was added to the cell culture and collected after 72 h, according to Melo et al. [21]. EVs were isolated using Total Exosome Isolation Reagent (Invitrogen) and quantified using Pierce™ BCA Protein Assay Kit (Thermo Fisher Scientific). The absorbance was read at 562 nm on an ELISA reader (BioTek). The EVs isolation was performed for all the cell lines using the same method above. The confirmation of EVs isolation was determined using the HCC1806 cells as a confirmatory measurement of exosome isolation. EVs size characterization was performed using the nanoparticle tracking analysis (NTA) in the Nano-Sight LM10 (Malvern Panalytical) instrument at Carlos Chagas Institute, Curitiba, PR, Brazil. Briefly, the samples were captured in 5 videos of 30 s, with the average used to assess the size distribution of EVs. The transmission electron microscopy (TEM) was also performed to check the HCC1806-EVs size and shape. Briefly, approximately 7 µg of the HCC1806-derived EVs were fixed on paraformaldehyde 4%, and added on a Formvar carbon-coated copper grid, followed by uranyl treatment. The EVs were then observed under a JEOL 1200EX II transmission electron microscope, 110V, available at the Electron Microscopy Center, Federal University of Paraná (UFPR), Curitiba, PR, Brazil. Western blot analysis was performed under non-reducing conditions, using primary antibodies specific for the proteins CD9 and CD63 (Invitrogen) (1:1000) and secondary antibody for horseradish peroxidase (HRP) (Invitrogen) (1:2000). The proteins were detected using the SuperSignal™ West Femto Maximum Sensitivity Substrate (Thermo Fisher Scientific) and captured with Amersham Imager 600 (GE Healthcare Life Science). Considering that these antibodies are commonly used as exosomal markers, but can also be present on other types of EVs, we adopted to use the general term EVs.

### Labeling assay

To confirm the interaction of the EVs isolated from the TNBC cells, as measured by the ones from the HCC1806 cells, with the recipient cells (MCF10A), a labeling assay using EVs from the HCC1806 labeled cells was performed. Briefly, the HCC1806 cells were labeled with PKH67 Green Fluorescent Cell Linker Kit for General Cell Membrane Labeling (Sigma-Aldrich), according to manufacturer’s instructions. The labeling efficiency was confirmed by analysis on the EVOS FL auto system (Invitrogen), after 48 h. The EV media were added, collected after 72 h, and the labeled HCC1806-EVs were isolated as described above. Approximately 10^4^ cells of MCF10a cells were treated with 0.02 µg/µl of labeled HCC1806-EVs and the interaction was evaluated after 48 h using the EVOS FL auto system.

### Cell viability and proliferation assays

Prior to the proliferation and cytotoxicity assays in the EVs derived cells, we assessed the cell viability upon EVs treatment in the HCC1806 cell line. Approximately 10^4^ HCC1806 cells were seeded in 96-wells plates and treated with 2 µg (0.02 µg/µl) of HCC1806-EVs. PBS was used as negative control. For the proliferation assays, 4 × 10^3^ MCF10A cells were seeded as described above, and treated with the HCC1806 established concentration of 0.02 µg/µl. The same EVs concentration was used for the other cell lines, with PBS used as negative control. The optimal EVs concentration for the other cell lines was not tested. The proliferation and cell viability curves were measured 48 h after treatment, using the Cell Titer 96® AQueous One Solution (Promega), according to manufacturer’s instructions. The absorbance was read at 490 nm on an ELISA reader (BioTek). All the assays were performed using biological and technical triplicates.

### Cytotoxic assays

For the cytotoxic assays, two chemotherapeutic agents, with distinct cellular mechanisms of actions, clinically used in breast cancer treatment were selected: Docetaxel (Taxotere) and Doxorubicin (Anthracycline) (Sigma-Aldrich). The cytotoxic responses were previously tested for each cell line (HCC1806 and MCF10A) for the identification of the IC50 values. Approximately 4 × 10^3^ cells of MCF10A were treated with 0.02 µg/µl of the HCC1806-isolated EVs and exposed to Docetaxel or Doxorubicin and their respective vehicles (no drug), or PBS (negative control). Two concentrations, based on the IC50 values, were chosen for each drug: 10 nM and 50 nM for Docetaxel and 100 nM and 500 nM for Doxorubicin, for the MCF10A and HCC1806, respectively. The MTT (3-(4,5-Dimethylthiazol-2-yl)-2,5-Diphenyltetrazolium Bromide) (Invitrogen) solution was prepared with media without FBS and used to evaluate the drug’s cytotoxicity. The absorbance was read at 562 nm on an ELISA reader (BioTek), after 48 h.

### Gene and miRNA expression analysis

In order to access the potential effects of the tumor-derived EVs in gene and miRNA expression of MCF10A cells, we performed gene and miRNA expression profiling. Total RNA was isolated from MCF10A cells treated with 0.02 µg/µl of HCC1806-derived EVs (MCF10A/HCC1806-EVs) and with PBS (MCF10A/PBS) (negative control), using TRIzol (Invitrogen). The experiments were performed in duplicates. Gene expression analysis was performed using the nCounter PanCancer Progression Panel (NanoString Tech), which consists of a panel of 770 genes associated with several steps of cancer progression. For the miRNA expression analysis, the nCounter Human v3 miRNA Expression Assay (NanoString) containing 799 probes that represents > 95% of all human miRBase reads was used. These assays were performed at the Genomics Shared Resource at the Ohio State University Comprehensive Cancer Center (OSUCCC). The raw data from both assays were processed by nSolver 4.0 software (NanoString) with the normalization performed with geometric mean for negative and positive controls, and standard parameters for CodeSet Content. The normalized data were then analyzed using the MultiExperiment Viewer software (MeV 4.9.0). Unsupervised and supervised hierarchical cluster analysis (HCL) was performed, using *t* test with Welch approximation to compare the cell lines groups. The hierarchical clusters were built using Pearson’s correlation coefficient and average linkage, adopting *p* < 0.05, based on permutation, with no corrections. The online tool Kyoto Encyclopedia of Genes and Genomes (KEGG) was used to identify the top signaling pathways potentially affected by gene and miRNA expression alterations. MiRNA target prediction was performed using Diana Tools microT-CDS [24, 27] and mirPath v.3 [38] and integrative analysis using mirTargetLink Human [10].

### Statistical analysis

Proliferation and cytotoxic assays data were normalized (D’Agostino & Pearson omnibus) (*p* > 0.05), and analyzed by paired *t* test, using GraphPad Prism v.6 (La Jolla). The Nanostring data analysis and normalization were performed using nSolver 4.0 software (NanoString). Heatmaps and cell type profiling analysis were generated by MeV 4.9.0 software. Results were considered statistically significant when *p* values < 0.05.

## Results

### Isolation and characterization of extracellular vesicles from breast cells

**Fig. 1:**
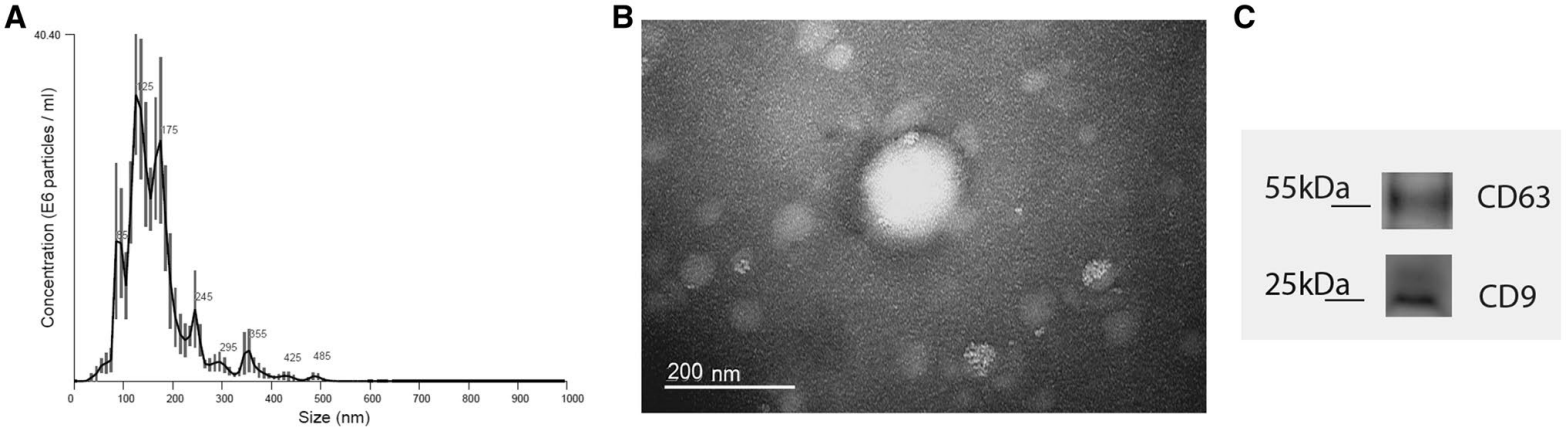
Characterization of EVs isolated from the culture media of the HCC1806 cells. **a** NTA analysis of HCC1806-EVs showing prominent peaks’ sizes between 100 and 200 nm. **b** TEM analysis showing a spheroid shape with size below 200 nm. **c** Western blot analysis for the exosomal markers, CD9 and CD63, and their respective protein sizes, showing positivity for both markers

EVs isolation from the culture media was performed for all cell lines using the precipitation method. The size distribution and shape of the isolated EVs was characterized for the HCC1806 cell only, as a confirmatory measurement of exosome isolation. Size distribution was accessed by NTA (Fig. 1a), showing a peak between 100 and 200 nm, with a mode of 129 nm. The TEM analysis showed a spheroid pattern, with a size below 200 nm (Fig. 1b), confirming the NTA results. The Western blot analysis showed positivity for CD9 and CD63 (Fig. 1c). These results confirmed that the HCC1806 cells were enriched with exosomal markers, within the expected exosomal size and shape.

### Fluorescence microscopy shows interaction of HCC1806-EVs and MCF10A cells

**Fig. 2:**
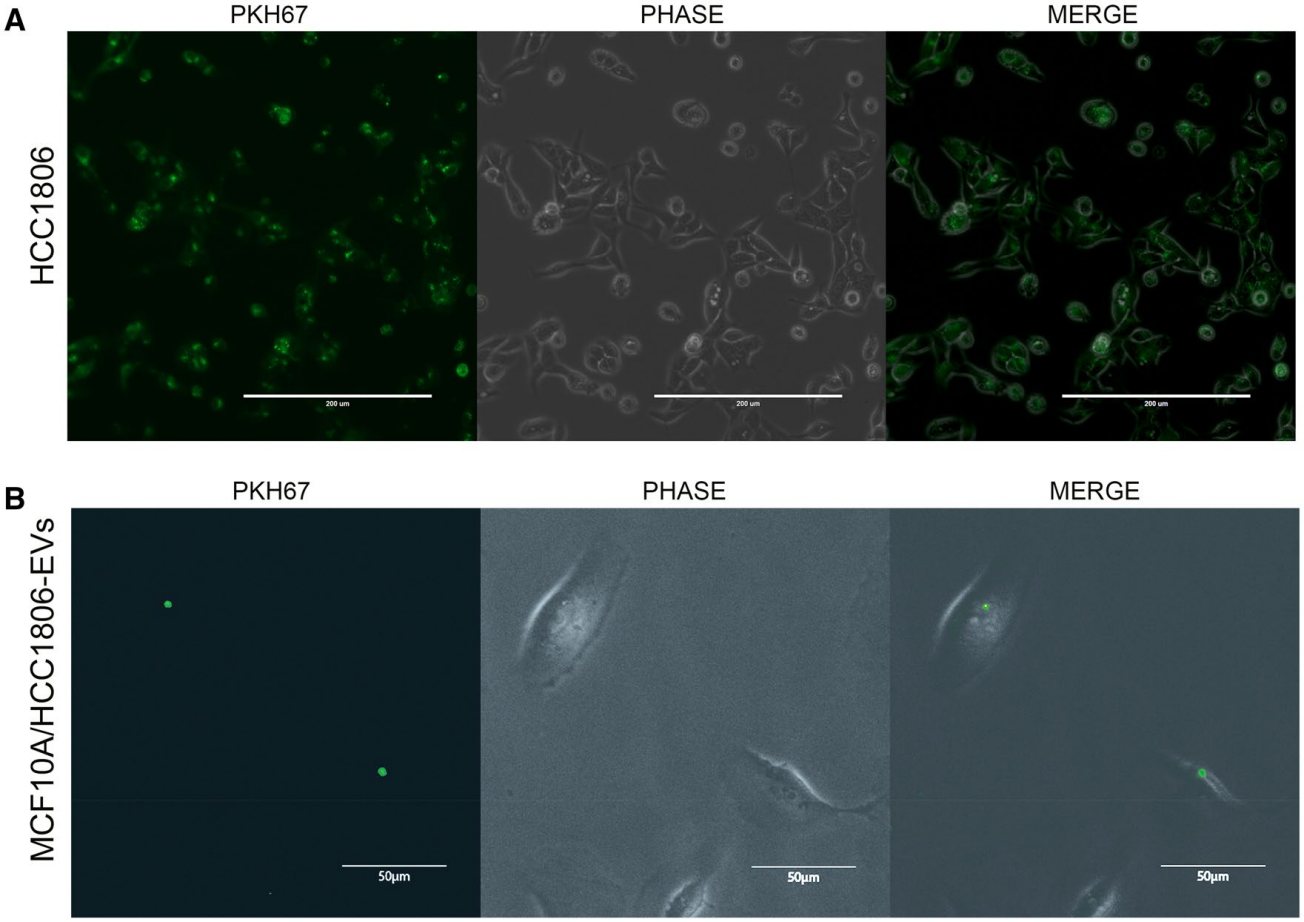
HCC1806-EVs labeling and interaction assays. **a** Fluorescence microscopy images of HCC1806 cells stained with PKH67 (left image), without the fluorescent filter (middle) and the overlap between the two images (right), after 48 h (scale bars: 200 nm). **b** Fluorescence microscopy images of MCF10A cells treated with PKH67-stained HCC1806-EVs (left image), without the fluorescent filter (phase) (middle) and the overlap between the two images (right), after 48 h (scale bars: 50 nm)

To confirm the interaction of the EVs isolated from the TNBC cells, a labeling assay using EVs from the HCC1806-labeled cells (Fig. 2a) was performed (this interaction was not tested for the MDA-MB-231 and/or MCF-7 cells). This assay showed the integration of the EVs isolated from the HCC1806 cells in the MCF10A cells (Fig. 2).

### HCC1806-EVs promote proliferation in MCF10A cells

**Fig. 3:**
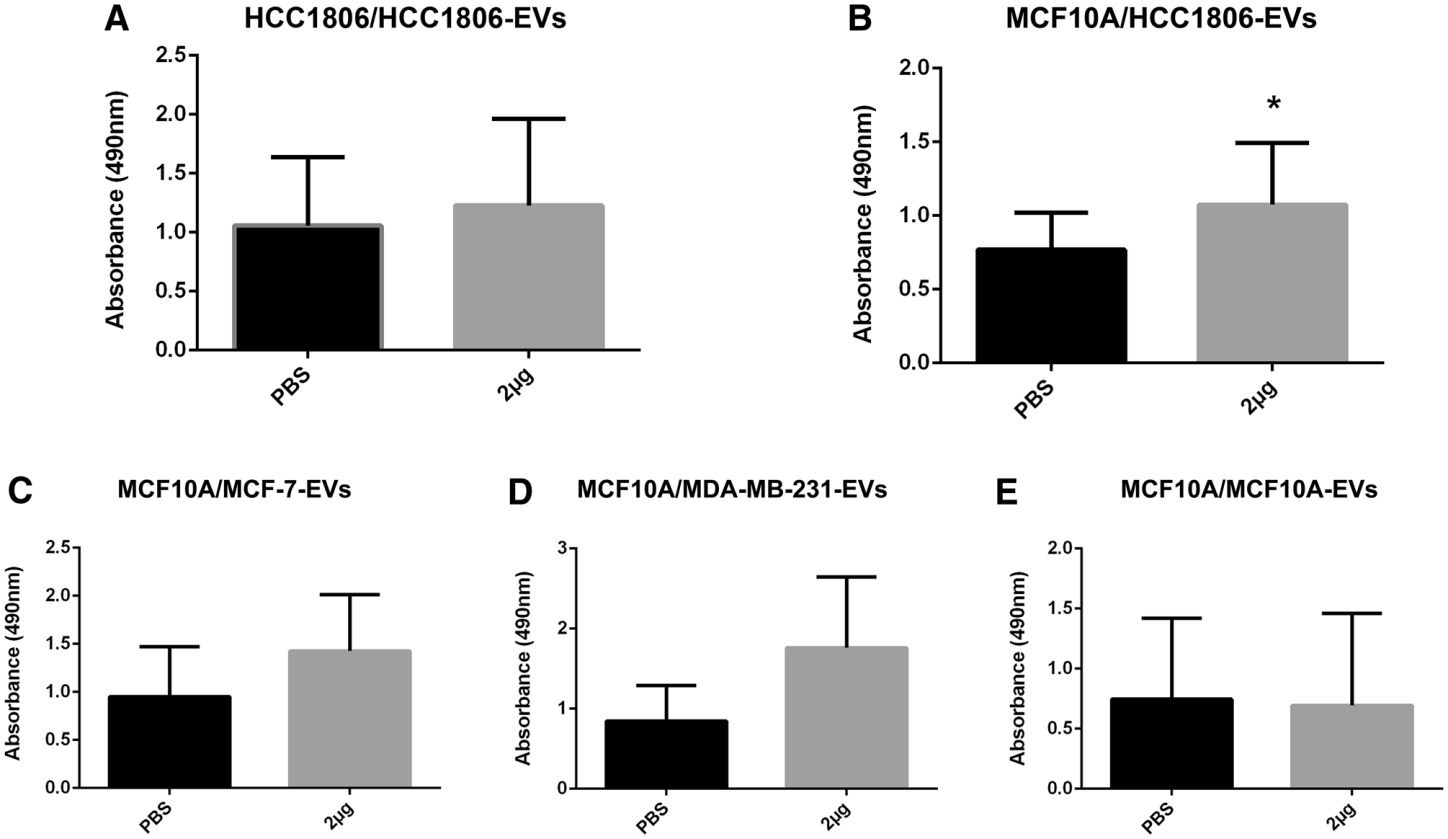
Effects of EVs treatment (2 µg) on the breast cell lines viability and proliferation in relation to the negative control (PBS). **a** No alteration on cell viability in the HCC1806 cells treated with 2 µg of HCC1806-EVs (*p* = 0.5112). **b** A significant proliferative effect on MCF10A cells treated with HCC1806-EVs (*p* = 0.0378). **c** No significant effect on cell proliferation in MCF10A cells treated with MCF-7 EVs (*p* = 0.1019), **d** MDA-MB-231 EVs (*p* = 0.0788), and **e** MCF10A EVs (*p* = 0.4269). Data are expressed as mean ± SD, *p* < 0.05 (*), in triplicate experiments

Prior to the proliferation assays, the toxicity potential of the EVs’ isolation precipitation method (Total Exosome Isolation Reagent) was determined. Cell viability was measured after 48 h on the HCC1806 cells after its treatment with 2 µg (0.02 µg/µl) of its own derived EVs. No changes in cell viability was observed with this concentration (Fig. 3a), confirming the non-toxicity of the precipitation method used. Treatment of the MCF-10A was then performed with EVs derived from the other breast cancer cell lines using the above concentration of EVs. A significant increase in cell proliferation was observed in the MCF10A cells treated with EVs from the HCC1806 (*p* < 0.05) when compared to the MCF10A treated with the negative control, PBS (Fig. 3b). No significant increase in cell proliferation was observed in the MCF10A cells that were treated with EVs from MCF-7 and MDA-MB-231 (Fig. 3c, d). To confirm that the proliferation increase was not due to the treatment of the MCF10A cells with EVs, irrespective of their tumorigenic potential, cell proliferation was assessed in these cells with their own EVs. No significant changes in cell proliferation were observed in relation to the control (Fig. 3e).

### HCC1806-EVs induce drug resistance in MCF10A cells

**Fig. 4:**
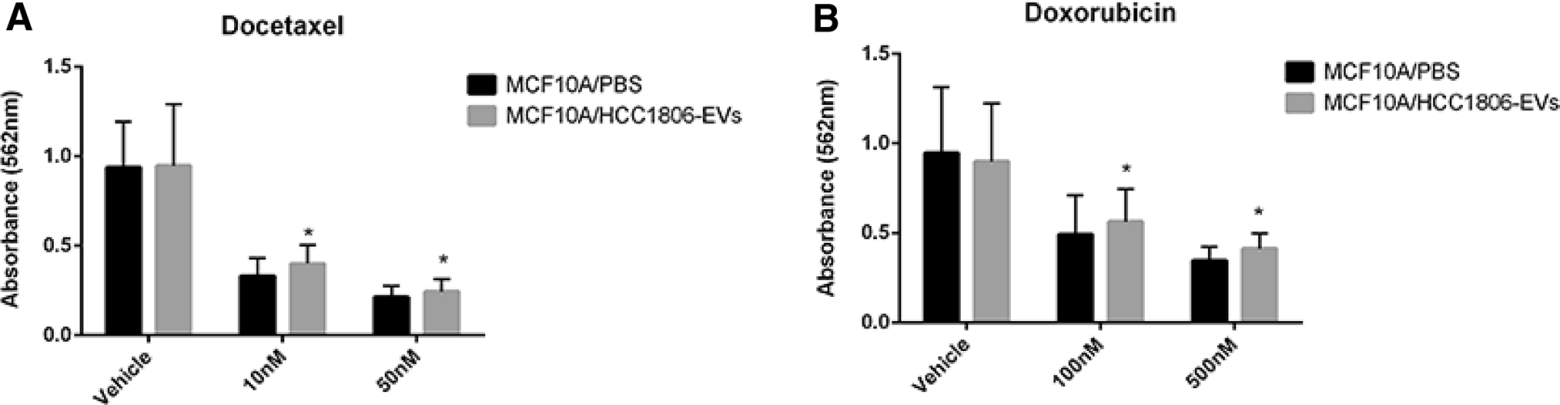
MCF10A cells treated with HCC1806-EVs and chemotherapeutic agents. **a** MCF10A cells treated with 2 µg of HCC1806-EVs caused reduction in cytotoxicity to Docetaxel, at 10 nM (*p* = 0.0208) and 50 nM (*p* = 0.0227), when compared to the control (vehicle). **b** MCF10A cells treatment with 2 µg of HCC1806-EVs caused reduction in cytotoxicity to Doxorubicin, at 100 nM (*p* = 0.0343) and 500 nM (*p* = 0.0121), when compared to the control (vehicle). Data are expressed as mean ± SD, *p* < 0.05 (*), in triplicate experiments

MCF10A cells were treated with HCC1806-EVs and PBS (negative control) and exposed to Docetaxel at 10 nM and 50 nM (Fig. 4a) and Doxorubicin at 100 nM and 500 nM (Fig. 4b) for 48 h. Cytotoxicity to these agents was not evaluated for the MCF10A cells treated with EVs derived from the other cell lines, considering that they did not cause any significant effect in the recipient cell proliferation. A statistically significant difference in cytotoxicity was observed in the MCF10A cells treated with HCC1806-EVs, for both treatments in relation to the vehicle (no drug), in the two concentrations tested for each agent. These results showed that MCF10A cells acquired resistance to these chemotherapeutic agents after treatment with HCC1806-EVs.

### HCC1806-EVs cause changes in the expression of genes associated with cell proliferation and apoptosis pathways in MCF10A cells

**Fig. 5:**
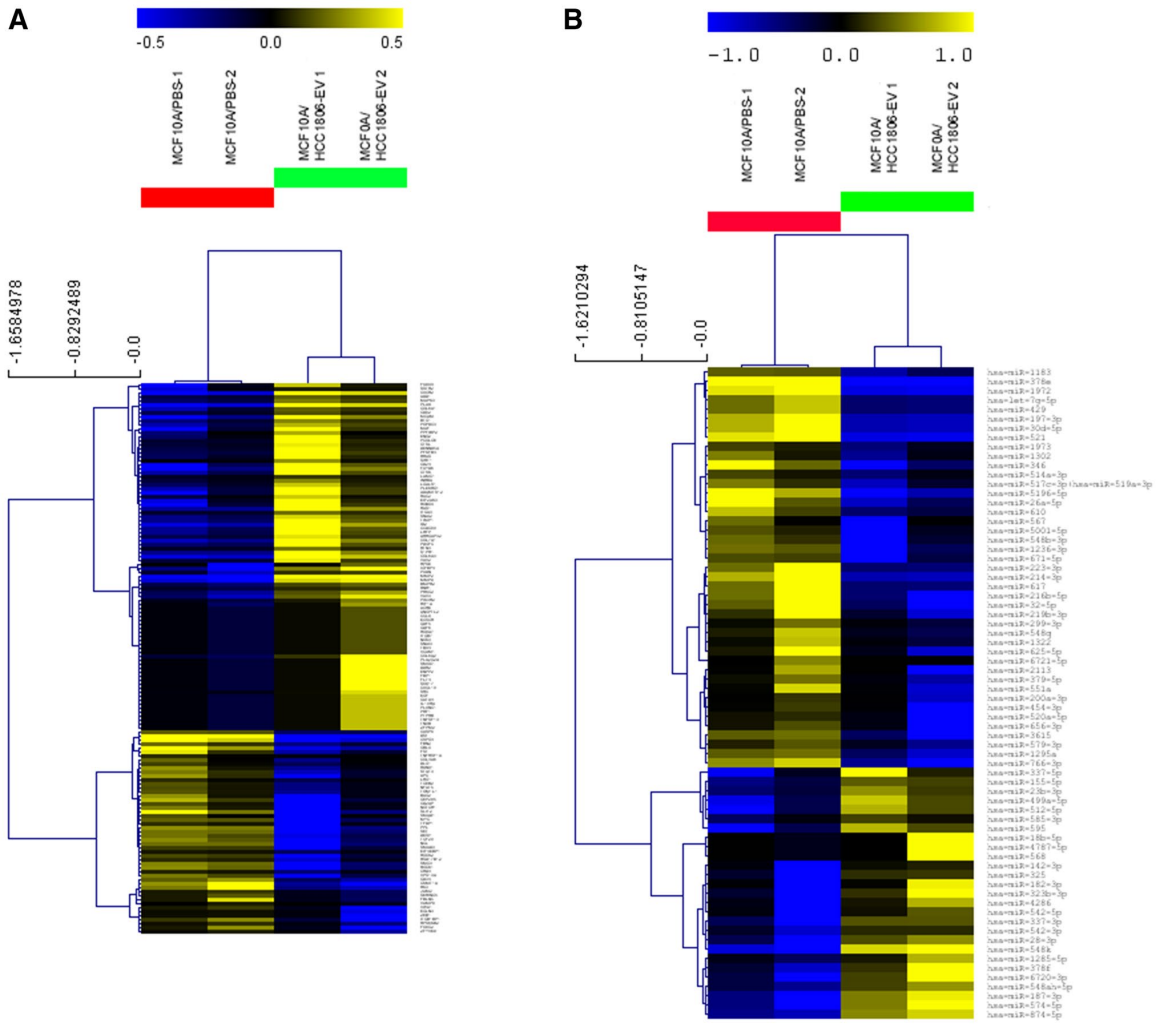
Supervised HCL analysis of DE genes (**a**) and miRNAs (**b**) observed among the MCF10A/HCC1806-EVs and control groups. Red and green lines indicate the control and the MCF10A/HCC1806-EVs groups, respectively. Up-regulated expressed genes and miRNAs are represented in yellow and down-regulated in blue. (MeV4.9.0, Pearson Correlation analysis, *p* < 0.05, duplicate experiments)

To evaluate the effects of HCC1806-EVs on the proliferation and cytotoxicity of MCF10A cells, a gene expression multiplexed cancer progression analysis was performed. (This analysis was not performed for the EVs derived from the other breast cancer cell lines, considering that no phenotypic changes were seen in the recipients’ MCF10A cells). Supervised HCL distinctly clustered the controls (MCF10A/PBS) and the treated (MCF10A/HCC1806-EVs) groups: 138 differentially expressed (DE) genes were found in this analysis, with 87 of them up-regulated and 51 down-regulated (Fig. 5a, Supplementary Table 1). According to the annotated functional categories of the genes composing the Progression panel, the observed DE genes were mainly related to extracellular matrix (ECM) layer (77 genes), tumor growth (59), angiogenesis (53), epithelial–mesenchymal transition (EMT) (50), tumor invasion (37), transcription factors (27), hypoxia (21), ECM remodeling (16), cancer metabolism (12), and metastasis (6). Considering that a given gene(s) could be involved in several of these processes, these categories may overlap. KEGG pathway analysis of the DE genes among the MCF10A/HCC1806-EVs and controls showed their involvement in pathways that included proteoglycans in cancer, PI3K/AKT, apoptosis, and MAPK signaling pathways (Table 1). The main genes related to the KEGG pathways and their respective fold changes, based on the comparison among the MCF10A/HCC1806-EVs and MCF10A/PBS groups, are listed in Table 2.

**Table 1:** Top fifteen KEGG pathways and corresponding number of the DE genes observed among the MCF10A/HCC1806-EVs and control groups (presented according to the number of genes affected)

KEGG #	Signaling pathways	# genes
ko05200	Pathways in cancer	17
ko05205	Proteoglycans in cancer	12
ko04390	Hippo signaling pathway	10
ko04060	Cytokine–cytokine receptor interaction	10
ko05206	MicroRNAs in cancer	10
ko04062	Chemokine signaling pathway	9
ko04350	TGF-beta signaling pathway	9
ko04510	Focal adhesion	9
ko04810	Regulation of actin cytoskeleton	9
ko04530	Tight junction	8
ko04151	PI3K-AKT signaling pathway	8
ko04066	HIF-1 signaling pathway	7
ko04010	MAPK signaling pathway	6
ko04210	Apoptosis	6
ko04014	Ras signaling pathway	5

**Table 2:** Main DE genes and their respective log2 fold changes and KEGG pathways, observed among the MCF10A/HCC1806-EVs and control groups

Gene	Log2 fold change	KEGG pathways
*CASP8*	0.78	Apoptosis
*CTSK*	1.49	Apoptosis
*CTSL*	1.27	Apoptosis
*CXCR2*	2.22	Chemokine signaling pathway
*EGF*	1.21	Apoptosis, HIF, PI3K-AKT, and MAPK signaling pathways
*EIF4EBP1*	0.90	HIF-1 signaling pathway
*FLT4*	1.28	PI3K-AKT signaling pathway
*HIF1A*	1.21	HIF-1 signaling pathway
*MAPK3*	1.28	MAPK signaling pathway
*MMP9*	2.04	Proteoglycans in cancer
*MYC*	0.46	MAPK and PI3K-AKT signaling pathways
*NOS3*	1.12	HIF-1, PI3K-AKT signaling pathways
*PDGFC*	1.46	PI3K-AKT signaling pathway
*PIK3R2*	1.18	PI3K-AKT signaling pathway
*PLAU*	1.96	Proteoglycans in cancer
*RRAS*	1.48	MAPK signaling pathway
*SRC*	1.20	PI3K-AKT signaling pathway
*TIMP1*	1.51	HIF signaling pathway
*TNFRSF1A*	0.95	Apoptosis
*TNFSF13*	1.21	PI3K-AKT signaling pathway

### HCC1806-EVs cause changes in the expression of miRNAs associated with pathways in cancer in MCF10A cells and present targets commonly affected by gene expression

MiRNA expression profiling was also performed in the MCF10A/HCC1806-EVs group in comparison to the control group. (As for gene expression profiling, this analysis was not performed for the EVs derived from the other breast cancer cell lines). Seventy DE miRNAs (Supplementary Table 2) distinctly clustered these two groups (Fig. 5b). Diana Tools mirPath v.3 analysis showed that the main pathways associated with the majority of these DE miRNAs were the ones related to cancer (Table 3), which could be one of the mechanisms by which the HCC1806-EVs treatment induced tumorigenic phenotypes in the MCF10A cells. An integrative analysis of the DE genes from the PanCancer Progressional Panel and the targets from the DE miRNAs was performed using the mirTargetLink software, showing ten common gene targets, which except for the ZFPM2, were down-regulated in the treated cells (Table 4). KEGG pathway analysis of these 10 genes showed their involvement in pathways in cancer, microRNAs in cancer and signaling pathways regulating pluripotency of stem cells, breast cancer, and proteoglycans in cancer. These results strength their participation as molecular mechanisms that could mediate the tumorigenic effects of the EVs in the treated MCF10A cells.

**Table 3:** Top fifteen KEGG pathways, their respective *p* value, number of targets, and DE miRNAs observed among the MCF10A/HCC1806-EVs and control groups (presented according to the number of DE miRNAs)

KEGG pathway	*p* value	# genes	# miRNAs
Pathways in cancer	2.49E−05	282	63
PI3K-Akt signaling pathway	0.003893	228	62
Ras signaling pathway	0.000135	159	59
Neurotrophin signaling pathway	0.000411	90	59
FoxO signaling pathway	0.00135	97	58
Focal adhesion	0.006598	142	58
cGMP-PKG signaling pathway	0.019202	113	58
Proteoglycans in cancer	2.47E−06	146	57
Sphingolipid signaling pathway	0.003494	82	57
Transcriptional misregulation in cancer	0.014825	117	57
Regulation of actin cytoskeleton	0.018333	144	57
Insulin signaling pathway	0.022425	97	57
Hippo signaling pathway	2.47E−06	112	56
Rap1 signaling pathway	0.0003	150	56

**Table 4:** Common genes and miRNA targets DE observed among the MCFA/HCC1806-EVs and control groups (presented by alphabetical order)

Target gene	miRNAs
*APC*	hsa-miR-142-3p^a^, hsa-miR-155-5p^a^
*HSD17B12*	hsa-miR-155-5p^a^
*MYC*	hsa-let-7 g-5p, hsa-miR-155-5p^a^, hsa-miR-26a-5p, hsa-miR-429
*NOTCH1*	hsa-miR-23b-3p^a^, hsa-miR-30d-5p
*ROCK2*	hsa-miR-142-3p^a^
*SMAD1*	hsa-miR-155-5p^a^, hsa-miR-26a-5p, hsa-miR-30d-5p
*SMAD3*	hsa-miR-155-5p^a^, hsa-miR-200a-3p
*STAT3*	hsa-miR-337-3p^a^
*ZEB1*	hsa-miR-1236-3p, hsa-miR-200a-3p, hsa-miR-23b-3p^a^, hsa-miR-429
*ZFPM2* ^a^	hsa-miR-200a-3p, hsa-miR-429

^a^Up-regulated

## Discussion

Triple-negative breast cancers (TNBCs) are usually characterized by an aggressive clinical behavior, which confers to the patients worse prognosis and short overall survival, when compared to hormone positive breast cancers [28, 35]. Given the high level of genetic heterogeneity of these tumors, the understanding of their molecular pathogenesis is of the utmost importance to the development of new therapy strategies with effective benefit to these patients.

Extracellular vesicles (EVs) present an important role on intercellular communication, an essential trait for the modulation of tumor microenvironment [31]. Previous studies [3, 40] have reported that the biological function and the content of EVs are dependent on the cell of origin. Interestingly, in our study, we observed that only the HCC1806, a TNBC cell line derived from a highly aggressive basaloid-TNBC, caused effects in the phenotype of the MCF10A cells. No effects were seen by the treatment with EVs from the MDA-MB-231 TNBC cell line, which is derived from a mesenchymal lineage. These results support the cell origin specificity of the EVs effect in mediating tumorigenesis in addition to the known distinct molecular signatures of the studied TNBC cell lines and their impact in proliferation rates and cytotoxicity response. However, we cannot rule out that phenotypic changes in MCFA cells could be induced if treated with higher concentrations of EVs from the MDA-MB-231 (and/or MCF-7) cells (not tested in this study).

The analysis of gene expression upon EVs treatment in recipient cells is a robust measurement of the effects of EVs and their impact in tumorigenesis [36]. In this study, we showed that treatment of the MCF10A cells with EVs derived from the HCC1806 cell line, led to changes in gene expression. A number of 138 DE genes were able to distinctly cluster the MCF10A/HCC1806-EVs and the control groups. KEGG signaling pathways analysis on these DE genes showed their involvement in several critical pathways associated with tumor progression, including pathways in cancer, PI3K/AKT, IL-8/CXCR2, ERK/MAPK, apoptosis, and HIF-1 signaling pathways. The PI3K/AKT signaling pathway is one of the most critical pathways involved in cancer, having a major role on cell proliferation and survival [37]. The *SRC* oncogene, involved in this pathway, was observed to be up-regulated in the MCFA10A/HCC1806-EV group when compared to the negative control group. The expression of this gene can be up-regulated by growth factors, such as the epidermal growth factor (EGF) [41], a gene that was also up-regulated in the MCF10A-treated cells. Other proliferative type genes up-regulated in the treated MCF10A/HCC1806-EVs group included the *FLT4, NOS3, PIK3R2*, and *PDGFC* genes. The Interleukin 8 (*IL-8*) gene and its receptor, *CXCR2*, have been described as markers of tumor progression, acting through repression of the *AKT1* gene on breast cancer cell lines [42]. Although in our study, the *AKT* gene was not observed DE among the groups, *CXCR2* was one of the most up-regulated genes observed on the MCFA10A/HCC1806-EV group. In addition, another gene ligand associated with *IL-8, TNFSF13* [19], was observed up-regulated in the EVs-treated cells. Another pathway that might be involved in the proliferative effects caused by HCC1806-EVs on the MCF10A cells was the MAPK pathway, also associated with activation of cell proliferation and survival [20]. The EGF expression and its up-regulation, might be responsible to the up-regulation of the *RRAS* [16], and *MAPK3* (also known as *ERK1*) expression, major effectors of this signaling pathway [6]. An interesting finding was the down-regulation of the *MYC* oncogene, commonly involved in both the PI3K/AKT and MAPK signaling pathways, indicating that the proliferative effect in the MCF10A treated cells probably occurred in a *MYC*-independent manner.

Another interesting trait of tumor-derived EVs are their capacity to induce changes in cytotoxicity response to chemotherapeutic agents [3]. Chen, et al. [3]. described that exosomes isolated from breast cancer cell lines resistant to Adriamycin and Docetaxel were responsible to transfer drug resistance to original sensitive cells. Corroborating with this study, our results showed a significant difference on the sensitivity of the MCF10A cells exposed to Docetaxel and Doxorubicin, when treated with HCC1806-EVs, demonstrating the capacity of these tumor EVs in inducing chemoresistance. Although these agents present distinct modes of action [7], both act on inducing apoptosis on cytotoxically treated cells. Consistent with these actions, we observed significant down-regulation in the expression of genes associated with apoptosis activation, which could be one of the mechanism by which the HCC1806-EVs induced chemoresistance in the MCF10A cells. The regulation of the expression of EGF and its downstream genes in the MAPK pathway, such as *RRAS* and *MAPK3*, have also been described as important inhibitors of apoptosis on breast cancer cells induced by chemotherapeutic agents [2]. As described above, these genes were up-regulated in the MCF10A-treated cells, and could therefore be inducing their acquired drug resistance phenotype. We also observed in the MCFA-treated cells, alterations in the expression of genes involved in the apoptosis extrinsic pathway, such as *TNFRSF1A* and *CASP8* [40], which were down-regulated in the MCF10A/HCC1806 EVs cells. Alterations in the expression of genes associated with angiogenesis, invasion, and metastasis were also observed in the MCF10A/HCC1806-EVs cells, including the *HIF1A, EIF4EBP1, EGF, NOS3, MMP9*, and *TIMP1* genes [36, 39, 40] that were DE in the MCF10A/HCC1806-EVs group, suggesting that the EVs might also be capable of inducing angiogenesis and metastasis.

miRNA profiling analysis was also performed in the treated MCF10A/HCC1806-EVS group in comparison to the control group, revealing 70 DE miRNAs. MiRNA target analysis revealed that ten of these miRNAs (miR-let7-5p, miR-23b-3p, miR-26a-5p, miR-30d-5p, miR-143-3p, miR-155-5p, miR-200a-3p, miR-337-3p, miR-429, and miR-1236) control genes that were also DE among the MCF10A/HCC1806-EVs and control groups, such as *APC, MYC, NOTCH1, SMAD1,3 STAT3*, and *ZEB1*. Among the up-regulated DE miRNAs, miR-155-5p, was the one that regulate the most number of the targets (five) that were DE in the treated cells: *APC*, *HSD17B12*, *MYC*, *SMAD1* and *SMAD3*. This miRNA, an oncomir described up-regulated in breast cancer in association with tumor initiation [8], is involved in the activation of the WNT signaling pathway, through down-regulation of *APC* [43], one of the DE genes found down-regulated among the groups studied. The other four targets of this miRNA (*HSD17B12*, *MYC, SMAD1, SMAD3)* were also observed down-regulated in the MCF10A/HCC1806-EVs group. Of most relevance to our study were the reports describing the expression of miR-155-5p in exosomes, and their role in conferring chemotherapy resistance and epithelial–mesenchymal transition (EMT) on breast cancer recipient cells [29]. In addition to miR-155-5p, mir-542-3p [18], let-7 and mir-28 [15], also included in the group of genes with concomitant gene and miRNA expression alterations, were previously associated with drug resistance in breast cancer.

## Conclusion

In conclusion, our results show that EVs isolated from the TNBC cells HCC1806 are capable of inducing proliferation and drug resistance on the non-tumorigenic MCF10A breast cells. Gene and miRNA profiling in the recipient cells suggest that these phenotypes could be mediated by changes in the expression of genes and miRNAs associated with proliferation, apoptosis, invasion, and migration. Additional functional studies to evaluate the role of the HCC1806-EVs on other cancer-related pathways are needed to comprehensively understand the unique mechanisms by which EVs impact TNBC pathogenesis.

## Electronic supplementary material

Below is the link to the electronic supplementary material.

Supplementary material 1 (DOC 211 KB)
