# Supplementary material for: Extracellular vesicles from triple-negative breast cancer cells promote proliferation and drug resistance in non-tumorigenic breast cells
Source: Breast Cancer Res Treat. 2018 Sep 1;172(3):713–23. doi: 10.1007/s10549-018-4925-5 (PMC6245099; doi:10.1007/s10549-018-4925-5)
Supplement: Supplementary file 1 — Supplementary material 1 (DOC 211 KB) [file 10549_2018_4925_MOESM1_ESM.doc]

**SUPPLEMENTARY MATERIAL**

**Supplementary table 1. Analysis of the nCounter PanProgression Panel. Fold change and progression annotated categories of differentially regulated genes (DEG) between MCF10a/HCC1806-EVs and MCF10a/PBS (control) groups.** Positive numbers indicate up-regulation on MCF10a/HCC1806-EVs when compared to the control.

| **Gene** | **Fold Change** | **Progression Categories** |
| --- | --- | --- |
| *ACHE* | 0.156083944 | ECM Layers, Tumor Growth, Tumor Invasion |
| *ADAMTS12* | 0.93831932 | ECM Layers, ECM Remodeling |
| *ADD1* | 0.27110863 | Angiogenesis, ECM Layers, Transcription Factor, Tumor Invasion |
| *ANGPTL2* | 0.156083944 | EMT |
| *APC* | -0.53370106 | Tumor Growth |
| *ARHGAP32* | 0.41012859 | ECM Layers, EMT |
| *BAG2* | -0.73370051 | EMT |
| *BMPR2* | 0.34542847 | ECM Layers, Tumor Growth |
| *BTG1* | 0.35495948 | Angiogenesis, Tumor Growth |
| *CASP8* | -0.35808515 | Angiogenesis, ECM Remodeling, Metastasis |
| *CBLC* | -1.07759343 | EMT, Tumor Growth |
| *CCDC80* | 0.82380867 | ECM Layers |
| *CCL8* | 0.156083944 | EMT |
| *CD24* | 0.83865834 | ECM Layers, EMT, Hypoxia, Tumor Growth, Tumor Invasion |
| *CD2AP* | -0.39296865 | ECM Layers, EMT, Tumor Growth |
| *CD34* | -0.49290455 | Angiogenesis, ECM Layers, Transcription Factor, Tumor Growth, Tumor Invasion |
| *CD82* | 0.231534955 | ECM Layers, Metastasis |
| *CEP295* | -1.04176139 | ECM Layers |
| *CHD4* | -0.198080065 | Transcription Factor |
| *CKMT1A* | -0.84192538 | EMT |
| *COL4A6* | -0.23547602 | ECM Layers, ECM Remodeling, Tumor Invasion |
| *COL6A1* | 0.38045406 | ECM Layers, ECM Remodeling, EMT, Tumor Growth, Tumor Invasion |
| *COL6A2* | 0.49751091 | ECM Layers, ECM Remodeling, EMT, Tumor Invasion |
| *COL6A3* | 1.34958174 | ECM Layers, ECM Remodeling, Tumor Invasion |
| *COL7A1* | 0.9645157 | ECM Layers, Tumor Invasion |
| *CSPG4* | -1.08975265 | Angiogenesis, ECM Layers |
| *CTSK* | 0.57943249 | EMT |
| *CTSL* | 0.3475194 | Metastasis |
| *CXCL10* | 0.713534954 | Angiogenesis, ECM Layers, Tumor Growth |
| *CXCR2* | 1.1546531 | ECM Layers, Tumor Growth, Tumor Invasion |
| *DDR2* | 0.363369104 | ECM Layers, EMT, Transcription Factor, Tumor Invasion |
| *DENND5A* | 0.49971986 | EMT |
| *DESI1* | -0.48245644 | EMT |
| *DLG1* | -0.162721634 | ECM Layers, Tumor Growth, Tumor Invasion |
| *DST* | -1.1661091 | ECM Layers, Tumor Growth, Tumor Invasion |
| *ECSCR* | 0.156083944 | Angiogenesis, ECM Layers |
| *EGF* | 0.266746044 | Angiogenesis, Cancer Metabolism, ECM Layers, Hypoxia, Tumor Growth |
| *EGLN3* | -0.260597944 | Cancer Metabolism, Hypoxia, Tumor Growth |
| *EIF2AK3* | 0.63947487 | Angiogenesis, Cancer Metabolism |
| *EIF4EBP1* | -0.153265956 | Cancer Metabolism, Hypoxia, Tumor Growth |
| *ENO2* | 0.69291496 | ECM Layers, Hypoxia |
| *ENPP2* | 0.363369104 | ECM Layers, EMT, Transcription Factor |
| *FBLN5* | -0.3615464 | Angiogenesis, ECM Layers, Tumor Invasion |
| *FBN2* | -0.34132433 | ECM Layers |
| *FBP1* | 0.363369104 | EMT, Tumor Growth |
| *FLT4* | 0.363369104 | Angiogenesis, ECM Layers, Tumor Growth |
| *FOXC2* | -0.6560933 | Angiogenesis, EMT, Transcription Factor, Tumor Growth, Tumor Invasion |
| *FOXO4* | 0.73032128 | Angiogenesis, Transcription Factor, Tumor Growth |
| *FST* | -0.47553778 | Tumor Growth |
| *FXYD6* | 1.00708342 | ECM Layers, EMT |
| *GATA4* | 0.266746044 | Angiogenesis, Transcription Factor, Tumor Growth |
| *GDF5* | 0.156083944 | Tumor Growth |
| *GDF6* | 0.156083944 | Tumor Growth |
| *HAS1* | 0.79145491 | ECM Layers, Tumor Invasion |
| *HIF1A* | 0.26739121 | Angiogenesis, Cancer Metabolism, ECM Layers, ECM Remodeling, EMT, Hypoxia, Transcription Factor, Tumor Growth |
| *HK3* | -0.6820973 | Cancer Metabolism, Hypoxia |
| *HOXA7* | 0.156083944 | Angiogenesis, Transcription Factor |
| *HPSE* | 0.429411884 | Angiogenesis, Tumor Growth, Tumor Invasion |
| *HSD17B12* | -0.310881134 | ECM Layers |
| *ID2* | 1.33305991 | Transcription Factor, Tumor Growth |
| *IGFBP4* | 1.1053319 | EMT, Tumor Growth |
| *IL10RA* | 0.266746044 | ECM Layers, EMT |
| *INHBA* | 0.23790717 | Tumor Growth |
| *ITGA3* | 0.24109936 | Angiogenesis, ECM Layers, ECM Remodeling, Tumor Invasion |
| *ITGB1BP1* | -0.60011648 | Angiogenesis |
| *ITGB7* | 0.156083944 | ECM Layers, ECM Remodeling, Tumor Invasion |
| *JAM2* | -0.20094776 | Angiogenesis, ECM Layers, EMT, Tumor Invasion |
| *LAMC1* | 0.1314044 | ECM Layers, ECM Remodeling, Tumor Growth, Tumor Invasion |
| *LGALS1* | 0.36577797 | ECM Layers, EMT |
| *LHFP* | 0.6972065 | ECM Layers, EMT |
| *LRG1* | -0.137287974 | Angiogenesis |
| *MAF* | 0.83311664 | EMT, Transcription Factor |
| *MAPK3* | 0.35973716 | Angiogenesis, Cancer Metabolism, Hypoxia, Tumor Growth, Tumor Invasion |
| *MCAM* | 0.82867837 | Angiogenesis, ECM Layers, Tumor Invasion |
| *MMP2* | 1.0877845 | Angiogenesis, ECM Layers, ECM Remodeling, EMT, Hypoxia |
| *MMP9* | 1.03515792 | ECM Layers, ECM Remodeling, EMT, Hypoxia, Tumor Growth |
| *MYC* | -1.11202094 | Cancer Metabolism, Transcription Factor, Tumor Growth |
| *NCL* | -0.7999773 | Angiogenesis |
| *NFAT5* | -0.24075532 | Angiogenesis, Transcription Factor |
| *NOS3* | 0.156083944 | Angiogenesis, ECM Layers, Hypoxia, Tumor Growth |
| *NOTCH1* | -0.62218523 | Angiogenesis, ECM Layers, EMT, Transcription Factor, Tumor Invasion |
| *PCOLCE* | 0.81417191 | ECM Layers, EMT |
| *PDGFC* | 0.55291749 | Angiogenesis, Cancer Metabolism, EMT, Tumor Growth |
| *PFKFB4* | 0.60600019 | Hypoxia |
| *PIK3R2* | 0.244987964 | Angiogenesis, Cancer Metabolism, Hypoxia, Tumor Growth |
| *PLA2G2A* | 0.448565254 | Angiogenesis |
| *PLAU* | 0.97278451 | Angiogenesis, ECM Layers, ECM Remodeling, Hypoxia, Tumor Growth, Tumor Invasion |
| *PLEKHO1* | 0.68505859 | ECM Layers, EMT |
| *PLXNC1* | 0.266746044 | ECM Layers, EMT, Tumor Invasion |
| *POPDC3* | 0.49510908 | ECM Layers, EMT |
| *PPFIBP2* | 0.28920984 | EMT |
| *PPL* | -0.76870037 | ECM Layers, EMT |
| *PRF1* | 0.266746044 | ECM Layers, EMT, Transcription Factor |
| *PRKCZ* | 0.46702432 | EMT, ECM Layers |
| *PTPRB* | 0.266746044 | Angiogenesis, ECM Layers, Tumor Invasion |
| *PXDN* | 0.358569146 | ECM Layers |
| *RAC2* | 0.304609536 | Angiogenesis, Cancer Metabolism, ECM Layers, Tumor Growth, Tumor Invasion |
| *RNH1* | 0.210126874 | Angiogenesis |
| *ROBO4* | 1.02329637 | Angiogenesis, ECM Layers |
| *ROCK1* | -0.70464563 | Angiogenesis, Tumor Growth, Tumor Invasion |
| *ROCK2* | -0.53060388 | Angiogenesis, ECM Layers, Tumor Growth, Tumor Invasion |
| *RPS6KB2* | -0.20282722 | Cancer Metabolism, Hypoxia, Tumor Growth |
| *RRAS* | 0.57113862 | Angiogenesis, ECM Layers |
| *RTN4* | 0.22565842 | Angiogenesis, ECM Layers, EMT |
| *RUNX1* | -0.33178353 | Angiogenesis, ECM Layers, Metastasis, Transcription Factor |
| *S1PR1* | 0.85243415 | Angiogenesis, ECM Layers, Tumor Growth, Tumor Invasion |
| *SERINC5* | -0.22476745 | ECM Layers, EMT |
| *SET* | -0.60405993 | Tumor Growth |
| *SIRT1* | 0.19432521 | Angiogenesis |
| *SLIT2* | -1.63535313 | Angiogenesis, ECM Layers, EMT, Tumor Growth |
| *SMAD1* | -0.538218495 | Transcription Factor, Tumor Growth |
| *SMAD3* | -0.55561542 | ECM Remodeling, Hypoxia, Transcription Factor, Tumor Growth |
| *SMC3* | -0.96358775 | ECM Layers, Tumor Growth |
| *SMOC1* | 0.448565254 | ECM Layers |
| *SNAI2* | 0.49797821 | EMT, Transcription Factor, Tumor Invasion |
| *SNAI3* | 0.156083944 | EMT, Transcription Factor |
| *SOX17* | 0.363369104 | Angiogenesis, Transcription Factor, Tumor Growth |
| *SRC* | 0.26263452 | Angiogenesis, ECM Layers, Tumor Growth, Tumor Invasion |
| *SSTR2* | 0.284957174 | ECM Layers, Tumor Growth |
| *STAT3* | -0.39542723 | ECM Layers, Hypoxia, Transcription Factor, Tumor Growth, Tumor Invasion |
| *TBX4* | 0.156083944 | Angiogenesis, Transcription Factor |
| *TCEB2* | -0.25603699 | Hypoxia |
| *TCF20* | -0.58026886 | Metastasis |
| *TFDP1* | -0.41684104 | Tumor Growth, Transcription Factor |
| *TIMP1* | 0.58672905 | Angiogenesis, ECM Layers, ECM Remodeling, Hypoxia, Metastasis, Tumor Growth |
| *TNFRSF1A* | -0.069474697 | Angiogenesis, Hypoxia |
| *TNFSF13* | 0.266746044 | ECM Layers, EMT, Tumor Growth |
| *TNXB* | 0.266746044 | ECM Layers, ECM Remodeling, Tumor Invasion |
| *TOM1L1* | -0.27307081 | EMT |
| *VAMP8* | -0.068664551 | ECM Layers, EMT |
| *VAV2* | 0.41403317 | Angiogenesis, ECM Layers, Tumor Growth |
| *VAV3* | 0.61110926 | Angiogenesis, ECM Layers, EMT, Tumor Growth, Tumor Invasion |
| *VCAM1* | 0.156083944 | Angiogenesis, ECM Layers, EMT, Hypoxia, Tumor Growth, Tumor Invasion |
| *VPS13A* | -0.60563398 | EMT |
| *VWA1* | -0.57092792 | ECM Layers |
| *ZEB1* | -0.337552306 | Tumor Growth, EMT, Transcription Factor |
| *ZFPM2* | 0.266746044 | Angiogenesis, EMT, Transcription Factor |
| *ZFYVE9* | -0.208251237 | Tumor Growth |

**Supplementary Table 2.**  **Analysis of the nCounter miRNA expression assay Fold Change Values and signaling pathways related to cancer of DE miRNAs between MCF10a/HCC1806-EVs and control groups.** Positive numbers indicate up-regulation on MCF10a/HCC1806-EVs when compared to the control.

| miRNA | Fold change | Signaling pathways related to cancer |
| --- | --- | --- |
| hsa-miR-548k | 2.62 | PI3K/AKT, Pathways in cancer, RAS, proteoglycan in cancer, transcriptional misregulation in cancer |
| hsa-miR-323b-3p | 2.02 | PI3K/AKT, Pathways in cancer, RAS, proteoglycan in cancer, transcriptional misregulation in cancer |
| hsa-miR-574-5p | 1.82 | PI3K/AKT, Pathways in cancer, RAS, proteoglycan in cancer, transcriptional misregulation in cancer |
| hsa-miR-595 | 1.71 |  |
| hsa-miR-187-3p | 1.69 | PI3K/AKT, Pathways in cancer, RAS |
| hsa-miR-6720-3p | 1.59 |  |
| hsa-miR-182-3p | 1.47 | PI3K/AKT |
| hsa-miR-325 | 1.42 |  |
| hsa-miR-337-5p | 1.4 | PI3K/AKT |
| hsa-miR-4787-5p | 1.34 | PI3K/AKT, Pathways in cancer, RAS, proteoglycan in cancer |
| hsa-miR-874-5p | 1.32 | PI3K/AKT, Pathways in cancer, RAS, proteoglycan in cancer, transcriptional misregulation in cancer |
| hsa-miR-378f | 1.31 | PI3K/AKT, Pathways in cancer, RAS, proteoglycan in cancer, transcriptional misregulation in cancer |
| hsa-miR-512-5p | 1.22 | PI3K/AKT, Pathways in cancer, RAS, proteoglycan in cancer, transcriptional misregulation in cancer |
| hsa-miR-28-3p | 1.19 | PI3K/AKT, Pathways in cancer, RAS, transcriptional misregulation in cancer |
| hsa-miR-499a-5p | 1.14 | PI3K/AKT, Pathways in cancer, RAS, proteoglycan in cancer, transcriptional misregulation in cancer |
| hsa-miR-337-3p | 1.09 | PI3K/AKT, Pathways in cancer, RAS, proteoglycan in cancer, transcriptional misregulation in cancer |
| hsa-miR-568 | 1.04 | PI3K/AKT, Pathways in cancer, RAS, proteoglycan in cancer, transcriptional misregulation in cancer |
| hsa-miR-4286 | 1.01 | PI3K/AKT, Pathways in cancer, RAS, proteoglycan in cancer, transcriptional misregulation in cancer |
| hsa-miR-142-3p | 0.95 | PI3K/AKT, Pathways in cancer, RAS, proteoglycan in cancer, transcriptional misregulation in cancer |
| hsa-miR-542-5p | 0.84 | PI3K/AKT, Pathways in cancer, RAS |
| hsa-miR-18b-5p | 0.83 | PI3K/AKT, Pathways in cancer, RAS, proteoglycan in cancer, transcriptional misregulation in cancer |
| hsa-miR-548ah-5p | 0.83 | PI3K/AKT, Pathways in cancer, RAS, proteoglycan in cancer, transcriptional misregulation in cancer |
| hsa-miR-23b-3p | 0.73 | PI3K/AKT, Pathways in cancer, RAS, proteoglycan in cancer, transcriptional misregulation in cancer |
| hsa-miR-155-5p | 0.69 | PI3K/AKT, Pathways in cancer, RAS, proteoglycan in cancer, transcriptional misregulation in cancer |
| hsa-miR-542-3p | 0.65 | PI3K/AKT, Pathways in cancer, RAS, proteoglycan in cancer |
| hsa-miR-1285-5p | 0.63 | PI3K/AKT, Pathways in cancer, RAS, proteoglycan in cancer, transcriptional misregulation in cancer |
| hsa-miR-585-3p | 0.4 |  |
| hsa-miR-6721-5p | -0.29 | PI3K/AKT, Pathways in cancer, RAS, proteoglycan in cancer, transcriptional misregulation in cancer |
| hsa-miR-299-3p | -0.39 | PI3K/AKT, Pathways in cancer, RAS, proteoglycan in cancer, transcriptional misregulation in cancer |
| hsa-miR-514a-3p | -0.44 | PI3K/AKT, Pathways in cancer |
| hsa-miR-1973 | -0.49 |  |
| hsa-miR-200a-3p | -0.5 | PI3K/AKT, Pathways in cancer, RAS, transcriptional misregulation in cancer |
| hsa-miR-1322 | -0.54 | PI3K/AKT, Pathways in cancer, RAS, proteoglycan in cancer, transcriptional misregulation in cancer |
| hsa-miR-579-3p | -0.58 | PI3K/AKT, Pathways in cancer, RAS, proteoglycan in cancer, transcriptional misregulation in cancer |
| hsa-miR-379-5p | -0.61 | PI3K/AKT, Pathways in cancer, RAS, proteoglycan in cancer, transcriptional misregulation in cancer |
| hsa-miR-1302 | -0.62 | PI3K/AKT, Pathways in cancer, RAS, proteoglycan in cancer, transcriptional misregulation in cancer |
| hsa-miR-548q | -0.66 | PI3K/AKT, Pathways in cancer, RAS, proteoglycan in cancer, transcriptional misregulation in cancer |
| hsa-miR-517c-3p+hsa-miR-519a-3p | -0.81 | Proteoglycan in cancer |
| hsa-miR-5001-5p | -0.83 | PI3K/AKT, Pathways in cancer, RAS, proteoglycan in cancer, transcriptional misregulation in cancer |
| hsa-miR-551a | -0.85 |  |
| hsa-miR-610 | -0.85 | PI3K/AKT, Pathways in cancer, RAS, proteoglycan in cancer, transcriptional misregulation in cancer |
| hsa-miR-1183 | -0.87 | PI3K/AKT, Pathways in cancer, RAS, proteoglycan in cancer, transcriptional misregulation in cancer |
| hsa-miR-567 | -0.93 | PI3K/AKT, Pathways in cancer, RAS, proteoglycan in cancer, transcriptional misregulation in cancer |
| hsa-miR-1295a | -0.99 | PI3K/AKT, transcriptional misregulation in cancer |
| hsa-let-7g-5p | -1.06 | PI3K/AKT, Pathways in cancer, RAS, proteoglycan in cancer, transcriptional misregulation in cancer |
| hsa-miR-2113 | -1.06 | PI3K/AKT, Pathways in cancer, RAS, proteoglycan in cancer, transcriptional misregulation in cancer |
| hsa-miR-429 | -1.06 | PI3K/AKT, Pathways in cancer, RAS, proteoglycan in cancer, transcriptional misregulation in cancer |
| hsa-miR-548b-3p | -1.14 | PI3K/AKT, Pathways in cancer, RAS, proteoglycan in cancer, transcriptional misregulation in cancer |
| hsa-miR-219b-3p | -1.18 | PI3K/AKT, Pathways in cancer, RAS, transcriptional misregulation in cancer |
| hsa-miR-3615 | -1.23 | PI3K/AKT, Pathways in cancer, RAS, proteoglycan in cancer, transcriptional misregulation in cancer |
| hsa-miR-617 | -1.3 | Proteoglycan in cancer, transcriptional misregulation in cancer |
| hsa-miR-671-5p | -1.31 | PI3K/AKT, Pathways in cancer, RAS, proteoglycan in cancer, transcriptional misregulation in cancer |
| hsa-miR-26a-5p | -1.34 | PI3K/AKT, Pathways in cancer, RAS, proteoglycan in cancer, transcriptional misregulation in cancer |
| hsa-miR-656-3p | -1.39 | PI3K/AKT, Pathways in cancer, RAS, proteoglycan in cancer, transcriptional misregulation in cancer |
| hsa-miR-223-3p | -1.48 | PI3K/AKT, Pathways in cancer, RAS, proteoglycan in cancer, transcriptional misregulation in cancer |
| hsa-miR-454-3p | -1.53 | PI3K/AKT, Pathways in cancer, RAS, proteoglycan in cancer, transcriptional misregulation in cancer |
| hsa-miR-1236-3p | -1.55 | PI3K/AKT, Pathways in cancer, RAS, proteoglycan in cancer, transcriptional misregulation in cancer |
| hsa-miR-32-5p | -1.56 | PI3K/AKT, Pathways in cancer, RAS, proteoglycan in cancer, transcriptional misregulation in cancer |
| hsa-miR-197-3p | -1.57 | PI3K/AKT, Pathways in cancer, RAS, proteoglycan in cancer, transcriptional misregulation in cancer |
| hsa-miR-30d-5p | -1.57 | PI3K/AKT, Pathways in cancer, RAS, proteoglycan in cancer, transcriptional misregulation in cancer |
| hsa-miR-216b-5p | -1.59 | PI3K/AKT, Pathways in cancer, RAS, proteoglycan in cancer, transcriptional misregulation in cancer |
| hsa-miR-625-5p | -1.6 | PI3K/AKT, Pathways in cancer, RAS, proteoglycan in cancer, transcriptional misregulation in cancer |
| hsa-miR-766-3p | -1.61 | PI3K/AKT, Pathways in cancer, RAS, proteoglycan in cancer, transcriptional misregulation in cancer |
| hsa-miR-214-3p | -1.89 | PI3K/AKT, Pathways in cancer, RAS, proteoglycan in cancer, transcriptional misregulation in cancer |
| hsa-miR-520a-5p | -1.91 | PI3K/AKT, Pathways in cancer, RAS, proteoglycan in cancer, transcriptional misregulation in cancer |
| hsa-miR-521 | -2.09 |  |
| hsa-miR-346 | -2.12 | PI3K/AKT, Pathways in cancer, RAS, proteoglycan in cancer, transcriptional misregulation in cancer |
| hsa-miR-1972 | -2.21 | PI3K/AKT, Pathways in cancer, RAS, proteoglycan in cancer, transcriptional misregulation in cancer |
| hsa-miR-5196-5p | -2.3 | PI3K/AKT, Pathways in cancer, RAS, proteoglycan in cancer, transcriptional misregulation in cancer |
| hsa-miR-378e | -5.1 | PI3K/AKT, Pathways in cancer, RAS, proteoglycan in cancer, transcriptional misregulation in cancer |
